# Fracture resistance of CAD/CAM milled versus direct hand-made interim laminate veneers

**DOI:** 10.1016/j.sdentj.2024.04.002

**Published:** 2024-04-16

**Authors:** Salahaldeen Abuhammoud, Banan Emtier, Chin-Chuan Fu, Silvia Rojas-Rueda, Carlos A. Jurado, Kelvin I. Afrashtehfar

**Affiliations:** a Department of Prosthodontics, University of Iowa College of Dentistry and Dental Clinics, Iowa City, IA, USA; b University of Iowa College of Dentistry and Dental Clinics, Iowa City, IA, USA; c Department of Restorative Sciences, University of Alabama at Birmingham School of Dentistry, Birmingham, AL, USA; d Division of Biomaterials, Department of Clinical and Community Sciences, School of Dentistry, University of Alabama Birmingham, Birmingham, AL, USA; e School of Dentistry, Pontifical Javerian University, Bogota, Colombia; f Division of Operative Dentistry, Department of General Dentistry, University of Tennesse Health Science Center College of Dentistry, Memphis, TN, USA; g Department of Reconstructive Dentistry and Gerodontology, School of Dental Medicine, University of Bern, Berne, BE, Switzerland; h Clinical Sciences Department, College of Dentistry, Ajman University, Ajman City, Emirate of Ajman, United Arab Emirates; i Private Practice Limited to Prosthodontics, Dubai, DU, United Arab Emirates; j Private Practice Limited to Prosthodontics, Abu Dhabi City, AZ, United Arab Emirates; k Department of Operative Dentistry, Periodontology and Preventive Dentistry, Rheinisch-Westfälische Technische Hochschule (RWTH) Aachen University Hospital, Aachen, Germany

**Keywords:** Dental veneers, Dental materials, Dental prosthesis design, Fracture resistance, CAD/CAM systems, Provisional restorations, Partial restorations, Cosmetic dentistry, Aesthetic dentistry, Laminate veneers, Temporary restorations, Indirect restorations, Direct restorations, Dental restorations, Dental prosthesis, Interim restorations

## Abstract

**Background:**

Comparative studies of interim veneer restorations crafted using subtractive computer-aided manufacturing (s-CAM) milling technology and traditional direct hand-made approaches are needed.

**Purpose:**

This comparative in vitro study evaluated the fracture resistance of two types of provisional veneer restorations for maxillary central incisors: milled (s-CAM) and traditional direct hand-made bis-acryl veneers.

**Materials and methods:**

Fifty maxillary right central incisor veneers (25 specimens per group) were fabricated and divided according to the fabrication method: (1) s-CAM milled (Structure CAD, VOCO Dental); and (2) hand-made (Protemp Plus, 3M). The restorations were cemented onto 3D-printed resin dies using temporary cement and subjected to 1000 cycles of thermal cycling between 5° and 55 °C. These restorations subsequently were subjected to compressive loading until fracture occurred. Images of the fractured samples were captured using a scanning electron microscope (SEM). Statistical analysis was performed using the one-way ANOVA test and the Mann-Whitney *U* test.

**Results:**

Significant differences (p < 0.001) in the fracture resistance were observed between the two groups. s-CAM milled interim veneers displayed higher fracture resistance values (439.60 ± 26 N) compared to the traditional method (149.15 ± 10 N).

**Conclusion:**

The manufacturing method significantly influences the fracture resistance of interim veneer restorations. s-CAM interim laminate veneer restorations for maxillary central incisors exhibit a fracture resistance superior to that of the traditional method using bis-acryl.

Clinical relevance

Clinicians should consider CAD/CAM milled veneers for scenarios demanding long-term interim restoration and the withstanding of high occlusal forces.

## 1 Introduction

In conservative dentistry, the goal is to preserve the tooth structure with ideal preparations, emphasizing tissue conservation, restoration success, uniformity, and minimal invasiveness (Gurel et al., 2013, Afrashtehfar and Afrashtehfar, 2016, Jurado et al., 2020). Among conservative restorative options, dental veneers hold a distinctive position. They serve as valuable restorations for optimal aesthetics and provide an alternative for tooth preservation, especially in younger adults where more invasive procedures like crown preparation might be considered (Cardoso et al., 2009, Alikhasi et al., 2022). Additionally, compared to the full-coverage crown preparations, dental veneer preparations preserve significant tooth structure, typically removing only 25 % of the coronal tooth structure (Edelhoff and Sorensen, 2002).

Innovative technologies, specifically computer-aided design and manufacturing (CAD/CAM), have revolutionized the fabrication of dental veneer restorations. Dental veneers are now produced using both novel CAD/CAM systems and traditional handcrafted methods, offering clinicians versatile options for diverse aesthetic needs (Jurado et al., 2023a, Villalobos-Tinoco et al., 2023). These contemporary approaches have expanded the treatment armamentarium and have consistently demonstrated the ability to deliver long-term, clinically successful results.

Aslan et al. (2019) analyzed 413 pressable lithium disilicate veneers and noted 98 % survival at 5 years, which slightly decreased over two decades to 87 %, with less than 4 % complication rate. Imburgia et al. (2021) reported a 98.83 % success rate for 1075 CAD/CAM veneers over 4 years. These studies demonstrated the longevity and predictability of lithium disilicate veneers in long-term dental restoration.

Upon completing veneer tooth preparations, the fabrication of provisional restorations is a critical step before finalizing the veneer restorations. These interim veneers serve to evaluate and refine the final restoration shape and shade, which are vital for ensuring aesthetic accuracy and patient satisfaction (Burke, 1993). Moreover, they offer several benefits, such as preventing hypersensitivity, reducing plaque buildup, decreasing caries risk, and preserving pulpal health. Provisional restorations also permit the maintenance of normal masticatory function and withstand physiological forces during chewing (Field and Wassell, 2023). Additionally, well-fitted provisional laminate veneers contribute to oral hygiene and periodontal health due to their anatomically appropriate design, which aids in effective cleaning (Jacques et al., 1999). It is noteworthy that these interim veneer restorations can be fabricated using either conventional techniques or modern CAD/CAM technology, with the choice influenced by the clinician's preference and technology availability.

An in-vitro study assessed the performance of CAD/CAM-produced provisional crowns for first premolars, such as VITA CAD Temp, PEEK, and Telio CAD, against traditional (handcrafted) Protemp crowns (Abdullah et al., 2016). The study measured marginal gaps and fracture strength and revealed that compared to the handcrafted method, CAD/CAM restorations had a smaller marginal gap and greater fracture resistance (Abdullah et al., 2016). However, research on interim veneer restorations using different fabrication techniques is still limited. Therefore, this study aimed to examine the fracture resistance of provisional laminate veneer restorations made via subtractive computer-aided manufacturing (s-CAM) and the conventional direct manual technique. The null hypothesis was that there is no significant difference in fracture resistance between veneers fabricated with these two techniques using the same material.

## 2 Materials and methods

### 2.1 Specimen preparation

This study used a typodont model of the maxillary right central incisor (1560 Dentoform, Columbia Dentoform, Lancaster, PA, USA). Three vinyl polysiloxane (VPS) putty guides (Splash Regular Set, Den-Mat Holdings LLC, Lompoc, CA, USA) were prepared. One guide was sectioned to assess incisal reduction, and another for facial reduction. Using these guides, the typodont tooth was prepared for a veneer restoration, involving 0.60 mm cervical and labial reduction, and 0.70 mm incisal reduction, as per the manufacturer's recommendations for lithium disilicate (IPS e.max CAD, Ivoclar Vivadent, Schaan, Liechtenstein).

The prepared tooth was digitally scanned with an intraoral scanner (Primescan, Dentsply Sirona, Charlotte, NC, USA), and the resultant stereolithography (STL) file was used to mill twenty-five veneer restorations (0.60 mm cervical and labial, 0.70 mm incisal) using a 5-axis milling machine (Ceramill Motion 2, Amman Girbach, Koblach, Austria) from the CAD/CAM composite (Structur CAD, VOCO GmbH, Cuxhaven, Germany). The fabrication materials for the interim veneer restorations are detailed in Table 1. The third putty guide facilitated the creation of twenty-five hand-made interim veneer restorations, each with 0.60 mm cervical and facial thickness, and 0.70 mm incisal thickness, using bis-acrylic composite (Protemp Plus, 3M, St. Paul, MN, USA) molded directly from the prepared typodont tooth.

**Table 1:** Description of materials used in the fabricating interim laminate veneers.

**Brand**	**Manufacture**	**Brand description**	**Components**
Structur CAD	VOCO GmbH, Cuxhaven, Germany.	CAD/CAM composite for temporary restorations.	Structur CAD contains 27 % inorganic fillers in a polymer matrix by weight.It contains methacrylates.
Protemp Plus	3 M, St. Paul, MN, USA	Bis-acrylic composite for temporary restorations.	Dimethacrylate (Bisema6) 45–55 %, amorphous silica, surface modified with propenoic acid, methyl, 3propyl ester and phenyltrimethoxy silane 20–30 %, 6-diisocyanatohexane with 2-methacryloyl-ethyl, 6-hydroxyhexanoate and 2-hydroxyethyl methacrylate (Desma) 10–15 %, and silane treated silica 5–10 %.

Fifty interim veneer restorations were cleansed in an ultrasonic bath (5300 Sweep Ultrasonic Cleaner, Quala Dental Products) with 90 % isopropyl alcohol for 5 min and allowed to dry at room temperature. These restorations were then cemented onto fifty resin dies, 3D-printed (Formlabs 3B, Formlabs Inc., Somerville, MA, USA) from a dental model resin (Model Resin, Formlabs, Somerville, MA, USA), using noneugenol temporary cement (Temp-Bond NE, Kerr Corporation, Brea, CA, USA).

### 2.2 Fracture strength test

**Fig. 1:**
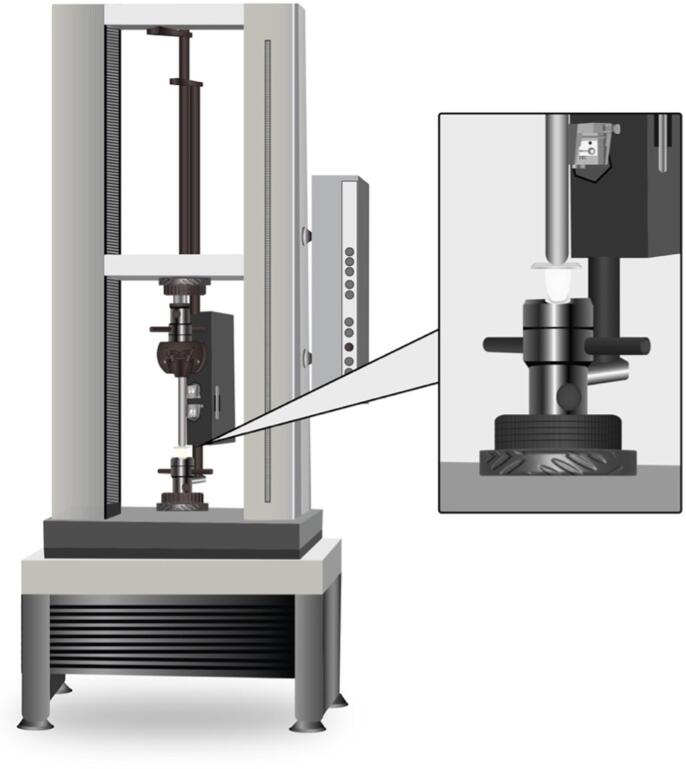
Illustration of the universal testing machine applying force to an interim veneer restoration during the fracture resistance test.

All cemented restorations underwent 1000 thermal cycles between 5 and 55 °C, with a 20-second dwell time. The veneers were then vertically loaded at the incisal edge with a flat applicator and a plastic layer for even force distribution. Fracture resistance was measured in Newtons using a ProLine ZwickRoell LP universal testing machine (Kennesaw, GA, USA) as shown in Fig. 1.

### 2.3 Fractographic analysis

Fractographic analysis was conducted on the fractured specimens using a Hitachi TM300 scanning electron microscope (Tokyo, Japan). The specimens were gold-coated for conductivity in a Sanyu Electron Quick Coater SC-701 sputter coater (Singapore, Singapore) before imaging at 10 kV. Crack numbers and lengths were evaluated in micrographs taken at 10× and 100× magnification.

### 2.4 Statistical analysis

Based on a G Power analysis from prior studies (Jurado et al., 2022, Jurado et al., 2023b), with α = 0.05 and power 0.8, the suggested sample size ranged from 9 to 35 for each group. Accordingly, in our study, 25 samples were selected per group. Statistical analysis was performed on the fracture resistance of interim veneer restorations for the maxillary right central incisor, comparing CAD/CAM milled to direct handmade methods. The analysis used SPSS Statistics version 27 (IBM, Chicago, IL, USA), employing one-way ANOVA and the Mann-Whitney *U* test to evaluate significant differences between the groups.

## 3 Results

### 3.1 Fracture resistance

The interim veneers for the maxillary right central incisor made with s-CAM and direct manual methods showed different fracture strengths (Table 2). The fabrication technique significantly affected the fracture resistance (p < .001), as analyzed by one-way ANOVA and the Mann-Whitney *U* test. The average fracture resistance of the CAD/CAM milled restorations was 470.88 N, while that of hand-made restorations was 148.76 N.

**Table 2:** Fracture resistance of interim veneer restorations fabricated by CAD/CAM milling and hand-made methods.

**Type of restoration**	**Number of samples**	**Fracture resistance values in Newtons**		
		**Mean (SE)**	**SD**	**Minimum and maximum values**
CAD/CAM milled interim veneers	25	439.60 (29.66)^a^	148.31	260.00, 899.00
Hand-made interim veneers	25	149.15 (10.72)^b^	53.61	42.00, 292.00

SE, standard error; SD, standard deviation.

Different lowercase indicates significant difference (p < 0.05).

### 3.2 Fractographic analysis

**Fig. 2:**
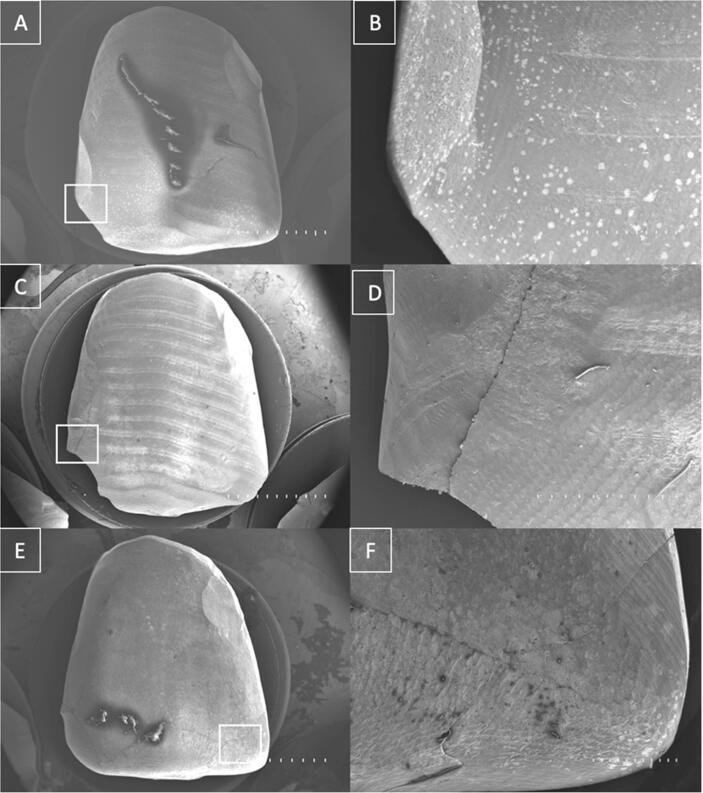
Scanning electron microscope images of three CAD/CAM milled interim veneers at 10x (A, C, and D) and 100x magnification (B, D, and F).

**Fig. 3:**
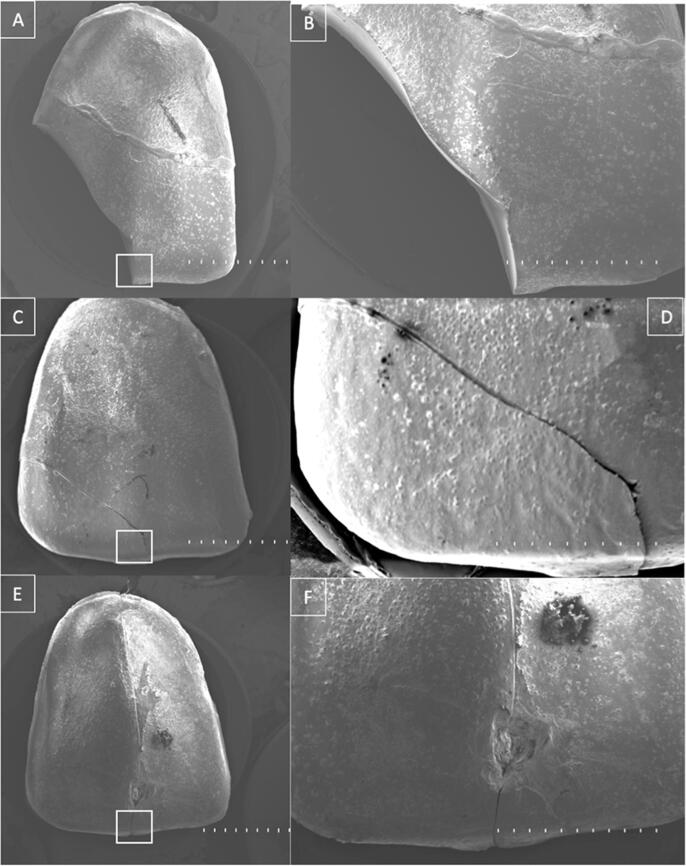
Scanning electron microscope images of three direct hand-made interim veneers at 10x (A, C, and D) and 100x magnification (B, D, and F).

Scanning electron microscopy (SEM) images (Fig. 2, Fig. 3) were used to characterize the fractured specimens. The CAD/CAM milled restorations had fewer, cleaner, and more defined cracks confined to the incisal edge. In contrast, the direct hand-made veneers showed more extensnive and irregular crack propagation throughout the body, resulting in more catastrophic failure.

## 4 Discussion

The central aim of this study was to assess the fracture resistance of provisional laminate veneer restorations, especially when comparing CAD/CAM milled restorations to those made by conventional hand-crafted methods. These results clearly refute the initial hypothesis that veneers made using different techniques, but the same material would exhibit comparable fracture resistance. Specifically, the study found that CAD/CAM milled veneers had a significantly greater mean fracture resistance of 470.88 N, while hand-made veneers exhibited a much lower mean fracture resistance of 148.76 N.

CAD/CAM dental technology has significantly advanced dentistry by streamlining the fabrication of restorations, reducing patient visits, and enhancing comfort and efficiency (Beuer et al., 2008). Secondary (Siqueira et al., 2021, Afrashtehfar et al., 2022) and primary studies (Sailer et al., 2017, Hashemi et al., 2022) endorse the advantages of intraoral scanning (IOS) over conventional impressions, emphasizing quicker procedures and preferred patient outcomes. Our study used a widely-used chairside scanner (Primescan, Dentsply Sirona), in line with the beneficial trends of CAD/CAM technology. While new dental technologies present challenges such as high initial costs and learning curves, with ongoing software updates adding to the complexity (Rekow and Thompson, 2005), they are counterbalanced by the efficacy of traditional methods. For instance, a retrospective study demonstrated the long-term reliability of hand-made lithium disilicate veneers, showing survival rates of 98 % at 5 years, 95 % at 10 years, 91 % at 15 years, and 87 % at 20 years (Aslam et al., 2019). Our study, recognizing the persistent use of conventional techniques, offers a thoughtful comparison of these time-tested approaches. The study aimed to fill the research gap on the fracture resistance of veneers by comparing CAD/CAM and manual methods. This is supported by evidence of the greater fracture resistance in CAD/CAM-fabricated full crowns (1243 N) and milled crowns (960 N) than in traditional hand-made crowns (558 N) (Alam et al., 2022). Another study highlighted the strength of CAD/CAM interim crowns for maxillary first premolars, which showed greater fracture resistance (910 N) than both 3D printed (720 N) and hand-made (620 N) crowns (Sakr et al., 2022).

### 4.1 Limitations of the study

Certain limitations of this study merit consideration. One potential limitation stems from the use of resin-printed dies in place of natural dentition, which may introduce some deviation from clinical reality. Resin-printed dies were selected for their ability to reduce confounding variables that are present with natural teeth. These include the difficulty of consistently identifying multiple caries-free central incisors, ensuring uniformity in tooth preparations, and managing natural teeth without introducing desiccation-induced artifacts. It is important to note that the use of printed dies over natural teeth is in line with the practices reported in prior studies (Lawson et al., 2019, Jurado et al., 2023b).

### 4.2 Recommendations for future research

Future research should aim to extend the scope of investigation to include interim veneer restorations for other anterior teeth, such as canines, to contribute to a more comprehensive understanding of these restorations in the aesthetic zone. Although our study did not examine the behavior of milling interim veneers in patients with parafunctional habits, further clinical investigations may elucidate the implications of using milled interim veneers for patients with high occlusal forces or parafunctional habits.

## 5 Conclusions

This study provides valuable insights into the fracture resistance of interim laminate veneers for maxillary central incisors by comparing those fabricated through indirect s-CAM milling and direct manual techniques. Considering the controlled environment and findings of this in vitro study, the following conclusions can be drawn:

1. Compared with their hand-made counterparts, s-CAM provisional laminate veneers exhibit significantly greater fracture resistance.
2. The findings suggest to clinicians that milled interim veneers may be a more resilient, and therefore, long-lasting treatment alternative.

## Ethical approval

Ethical approval was not required for this article as no human participants or animals were involved in the study.

## Declaration of competing interest

The authors declare that they have no known competing financial interests or personal relationships that could have appeared to influence the work reported in this paper.
